# The Effect of Dietary Nitrate on the Oral Microbiome and Salivary Biomarkers in Individuals with High Blood Pressure

**DOI:** 10.1016/j.tjnut.2024.07.002

**Published:** 2024-07-16

**Authors:** Lisa du Toit, Michaela L Sundqvist, Alvaro Redondo-Rio, Zöe Brookes, Patricia Casas-Agustench, Mary Hickson, Alicia Benavente, Gemma Montagut, Eddie Weitzberg, Toni Gabaldón, Jon O Lundberg, Raul Bescos

**Affiliations:** 1 School of Health Professions, Faculty of Health, University of Plymouth, Plymouth, United Kingdom; 2 Swedish School of Sport and Health Sciences, Department of Physiology, Nutrition and Biomechanics, Stockholm, Sweden; 3 Institute for Research in Biomedicine (IRB Barcelona), Barcelona, Spain; 4 Barcelona Supercomputing Centre (BSC-CNS), Barcelona, Spain; 5 Peninsula Dental School, Faculty of Health, University of Plymouth, Plymouth, United Kingdom; 6 Pharmacology and Physiology Department, Karolinska Institute, Stockholm, Sweden; 7 Catalan Institution for Research and Advanced Studies (ICREA), Barcelona, Spain; 8 CIBER de Enfermedades Infecciosas, Instituto de Salud Carlos III, Barcelona, Spain

**Keywords:** nitrate, nitrite, microbiome, nitric oxide, oral health, leafy green vegetables

## Abstract

**Background:**

Green leafy vegetables (GLV) contain inorganic nitrate, an anion with potential prebiotic effects on the oral microbiome. However, it remains unclear whether GLV and pharmacological supplementation [potassium nitrate (PN)] with a nitrate salt induce similar effects on the oral microbiome.

**Objectives:**

This study aimed to compare the effect of GLV with PN supplementation on the oral microbiome composition and salivary biomarkers in individuals with high blood pressure.

**Methods:**

Seventy individuals were randomly allocated to 3 different groups to follow a 5-wk dietary intervention. Group 1 consumed 300 mg/d of nitrate in form of GLV. Group 2 consumed pills with 300 mg/d of PN and low-nitrate vegetables. Group 3 consumed pills with potassium chloride (placebo: PLAC) and low-nitrate vegetables. The oral microbiome composition and salivary biomarkers of oral health were analyzed before and after the dietary intervention.

**Results:**

The GLV and PN groups showed similar microbial changes, probably nitrate-dependent, including an increase in the abundance of *Neisseria*, *Capnocytophaga, Campylobacter* species, and a decrease in *Veillonella*, *Megasphaera*, *Actinomyces*, and *Eubacterium* species after the treatment. Increased abundance of *Rothia species,* and reduced abundance of *Streptococcus, Prevotella*, *Actinomyces*, and *Mogibacterium* species were observed in the GLV group, which could be nitrate-independent. GLV and PN treatments increased salivary pH, but only GLV treatment showed an increase in the salivary buffering capacity and a reduction of lactate.

**Conclusion:**

The combination of nitrate-dependent and nitrate-independent microbial changes in the GLV group has a stronger effect to potentially improve oral health biomarkers compared with PN.

## Introduction

Dietary nitrate is a bioactive compound that serves as a precursor of nitric oxide, a crucial signaling molecule involved in multiple physiological processes [1]. Green leafy vegetables (GLV) and beetroot are the main dietary source of this anion [2]. Recent studies using concentrated beetroot juice indicate that dietary nitrate may have a prebiotic effect on the oral microbiome [[3], [4], [5], [6]], including an increase in the abundance of *Rothia* and *Neisseria* species, and a decrease in *Prevotella* and *Veillonella* species. [[3], [4], [5], [6]]. *Veillonella* and *Rothia* are significant contributors to nitrate reduction into nitrite in the oral cavity [7,8]. The ability of these bacteria to produce nitrite and to metabolize lactic acid as a carbon source during denitrification seems to be an important mechanism to regulate salivary pH [7,9]. Maintaining an optimal salivary pH is crucial for preserving oral health, as levels below 7 can lead to enamel demineralization, increasing risk of dental erosion, cavities, and the formation of microbial biofilms [10] The consumption of nitrate in the form of lettuce juice has also been associated with reduced gingival inflammation in patients and rats with periodontal disease [6,11], suggesting benefits for managing periodontal disease.

Research on dietary nitrate over the past decade has primarily focused on its potential ergogenic and hypotensive effects. From the second perspective, a substantial body of literature suggests that dietary nitrate effectively reduces blood pressure (BP), particularly in healthy individuals [[12], [13], [14], [15], [16], [17]]. However, this effect appears to be more limited in hypertensive individuals [[18], [19], [20], [21], [22]]. The Diet and Nitric Oxide (DINO) study, the largest clinical trial to date on dietary nitrate, did not find a reduction in BP in individuals with high BP after a 5-wk dietary intervention with nitrate [20]. Currently, explaining the discrepancies regarding the hypotensive effect of dietary nitrate is difficult. Although bacterial nitrate reduction does not seem to be a limiting factor, as suggested by the DINO study, it is important to consider the potential impact of poor oral health on nitrate and nitrite metabolism. Observational studies have highlighted a potential association between periodontal disease and hypertension [[23], [24], [25], [26]]. However, the nature of this relationship, whether causal or casual, is not fully understood.

Oral nitrate-reducing bacteria have emerged as a potential link between periodontal disease and hypertension. From this perspective, a positive association between the abundance of oral nitrate-reducing species and BP levels has been established in healthy individuals, but this has not been confirmed in hypertensive individuals [27]. Additionally, a recent study found that patients with periodontal disease exhibited lower abundance of oral nitrate-reducing bacteria, although BP levels were not considered in this study [28]. In rodents, it has been shown that dietary nitrate can partially reverse pre-existing periodontitis and endothelial dysfunction [11].

An important question that remains to be addressed is whether different sources of dietary nitrate, GLV or pharmacological salts [potassium nitrate: (PN)], have the same effect on the oral microbiome. Thus, the main aim of this study was to analyze the effect of GLV and PN on the composition of the oral microbiome and salivary biomarkers of oral health. Additionally, we analyzed potential associations between shifts in oral bacteria and alterations in BP levels after each dietary intervention. We hypothesized that dietary nitrate in both forms (GLV and PN) would induce similar changes, increasing the abundance of *Rothia* and *Neisseria* species while decreasing *Prevotella* and *Veillonella* species, thereby increasing the abundance of oral nitrate-reducing species (nitrate-dependent response). A higher abundance of nitrate-reducing bacteria would be associated with a greater reduction in BP levels. Additionally, we hypothesized that GLV would have a stronger effect on altering the composition of the oral microbiome (nitrate-independent) because of the presence of other bioactive molecules (e.g., polyphenols, vitamins). This effect would lead to beneficial alterations in salivary pH, buffering capacity, and lactate levels, thereby enhancing resilience against periodontal disease.

## Methods

### Participants

In this study we analyzed saliva samples collected from a subgroup of individuals from the DINO study (registered at clinicaltrials.gov as NCT02916615) [20]. The study was approved by the local research Ethics Committee in Stockholm (Sweden) and the Human Research Ethics and Integrity Committee of the University of Plymouth. Briefly, participants were 50 to 70-y-old women and men with a clinical systolic BP (SBP) of 130 to 159 mmHg and diastolic BP (DBP) <110 mmHg, recruited in Stockholm (Sweden). Exclusion criteria included a cardiovascular event within the previous 6 mo, change in dose of antihypertensive medication within the previous 2 mo, or use of organic nitrates or proton pump inhibitors. Also, persons who were vegetarian, vegan, or allergic to any of the vegetables included in the study protocol could not be enrolled. Individuals were allowed to continue taking their antihypertensive medication during the trial.

### Study design

The study design is described in detail in Sundqvist et al. [20]. Briefly, the study used a randomized, placebo-controlled, and single-blinded design and comprised 3 phases: screening, run-in, and intervention.

The screening involved a telephone interview and 2 clinic visits. During each clinic visit, SBP and DBP were measured in triplicate using an automatic Omron 110-IT BP monitor (Omron Corporation), after 5 min of rest in a seated position. The mean of the second and third readings was used for analysis. Individuals with a clinical SBP between 130 and 159 mmHg and DBP <110 mmHg at the second clinic visit were invited to participate in the study.

The run-in phase took 2 wk. All the participants were provided with low-nitrate vegetables (carrots, bell peppers, cherry tomatoes, and sweet corn) to be consumed twice daily alongside main meals (250 g/d). At the end of this period, fasting blood and saliva samples were collected and SBP and DBP were measured again.

The intervention phase started with randomization to allocate the participants within 1 of 3 groups: *1*) low-nitrate vegetables (<20 mg nitrate/d) + placebo pills (potassium chloride) (PLAC); *2*) low-nitrate vegetables + PN pills (300 mg nitrate/d) (PN); *3*) GLV (300 mg nitrate/d) + placebo pills. Participants were instructed to take a pill alongside their designated vegetables twice daily for 5 wk. The groups receiving low-nitrate vegetables were double-blinded, whereas the GLV group was single-blinded, meaning the investigators were aware that they were given a placebo pill. The exact amount of vegetables given to the patients was adjusted to ensure a daily intake of 300 mg (2 × 150 mg) nitrate. The nitrate dose of 300 mg/d was chosen based on previous research showing a reduction in BP after providing a similar amount of dietary nitrate in form of beetroot juice to hypertensive individuals over 4 wk [29]. Furthermore, this amount of nitrate was chosen considering what can be readily achievable from a normal diet.

The amount (weight) of vegetables was matched in the 3 groups and the nitrate content in the GLV was measured 4 times each year. The DINO study was conducted over a period of 5 y, during which the nitrate content in vegetables was measured multiple times throughout the year to ensure an intake equivalent to 300 mg nitrate [30]. Estimated nutritional content for the different vegetable groups was also calculated using databases. The daily portions of high-nitrate vegetables and low-nitrate vegetables had only minor nutritional differences. Participants visited the clinic each week for measuring the BP and collecting the vegetables. Saliva and blood samples were collected in the final week.

### Analyses

#### Sample preparation

Whole saliva samples was centrifuged (5,668×*g*; 10 min; 4°C). The supernatant was collected into a sterile Eppendorf tube and stored at −80°C for biochemistry analysis. The pellet in the bottom of the tube was also stored at −80°C to analyze the oral microbiome profile.

#### Saliva and plasma nitrate and nitrite

Saliva and plasma nitrate and nitrite were measured using an HPLC system (ENO-20; EICOM) as previously described [31].

#### Saliva pH and buffering capacity

Saliva pH was measured using a single electrode digital pH meter (Lutron Electronic Enterprise Co Ltd., Model PH-208) that was calibrated following the manufacturer’s instructions.

The saliva buffering capacity was measured mixing 100 μL of saliva with 300 μL of HCl (3.3 mmol/L) and shaken for 20 min. Then, salivary pH was measured using the single electrode digital pH meter.

#### Saliva glucose, lactate, and ammonia

Saliva glucose and lactate levels were analyzed in duplicate using a biochemistry analyzer (YSI 2500 Stat Plus, YSI Life Sciences) that was calibrated using the manufacturer instructions. Ammonia levels were measured using an assay kit (Merck Life Science AA0100). The standards were created according to manufacturer instructions. Ten µL of L-glutamate dehydrogenase was added to 100 µL of sample and then it was incubated for an additional 5 min before measuring the final absorbance at 340 nm.

#### Oral microbiome

##### DNA extraction and sequencing

Saliva pellets were used for the analysis of the microbiome. DNA extraction was performed with the ZymoBIOMICS 96 MagBead DNA kit (ref #D4302, ZymoResearch), using the FastPrep-96 and 32-PurePrep (MolGen). Sequencing was performed in the Illumina MiSeq using v3 chemistry at the Genomics facility of the Centre for Genomic Regulation. For additional details about the extraction and sequencing of the oral microbiome, refer to Supplemental Data 1.

### Statistical analysis

Results are presented as mean ± SEM. Normal distribution of the sample was assessed using the Shapiro–Wilk test. Between-group differences at baseline were analyzed using 1-way ANOVA followed by Tukey’s post hoc analysis when data were normally distributed or by the Kruskal–Wallis H test if data were non-normally distributed. Within-group comparison was analyzed with 2-factor repeated measures ANOVA (time × treatment). The association between BP levels and salivary nitrate/nitrite levels with other salivary biomarkers at baseline was analyzed using 2-tailed Spearman’s rank correlation analyses (95% confidence). Analysis was carried out using the SPSS software (SPSS Statistics, IBM Version 24) and statistical significance was determined as *P* < 0.05.

Microbial community analyses were performed with R v4.3.1. Dada2 v1.12.1 was used for quality filtering, with parameters “truncLen = c(270, 225), trimLeft = 10 minLen = 50, maxEE = 8, maxN = 0.” Dada2 was also used for amplicon sequence variants (ASV) clustering, chimera removal, and taxonomic assignment of ASVs with database SILVA nr99 v138.1. Phyloseq v1.44 was used to analyze the abundance tables. Abundance tables were 0-replaced using the count zero multiplicative (CZM) method in zCompositions v1.4.0.1 and then CLR-transformed with CoDaSeq v0.99.6. Differential abundance analyses were performed using mixed-effect linear models with lmer4 v1.1.33. When comparing paired samples, the subject was added as a random effect. *P* values for the multiple tests were adjusted using the Benjamini–Hochberg correction (false discorvery rate [FDR] correction). Nitrate-reducing species and genera were identified following the classification from Goh et al. [27] and species *Neisseria perflava* was added to the list as well. For additional details about the statistics of the oral microbiome, refer to Supplemental Data 2.

## Results

### Characteristic of study participants

Table 1 shows the baseline characteristics of the individuals. There were no differences (*P* > 0.05) in age, body weight, BMI (kg/m^2^), heart rate (HR), and BP. No changes were observed in body weight (PLAC: 82.4 ± 2.8; PN: 75.9 ± 3.5; GLV: 77.0 ± 2.8 kg), and BMI (PLAC: 26.3 ± 0.8; PN: 25.2 ± 0.8; GLV: 26.1 ± 0.7 kg/m^2^) after any of the dietary interventions compared with baseline (Table 1).

**Table 1:** Baseline characteristics of the participants.^1^

	All (*n* =70)	PLAC (*n* = 23)	PN (*n* = 23)	GLV (*n* = 24)
Age (y/o)	61.6 ± 0.6	61.3 ± 1.1	59.9 ± 1.0	63.6 ± 1.1
Gender (m/f)	36/34	16/7	9/14	11/13
Weight (kg)	78.3 ± 1.7	82.5 ± 2.8	75.8 ± 3.5	76.8 ± 2.8
BMI (kg/m^2^)	25.8 ± 0.4	26.4 ± 0.8	25.1 ± 0.8	26.0 ± 0.6
Heart rate (bpm)	67 ± 1	68 ± 2	66 ± 2	66 ± 2
SBP (mmHg)	131 ± 2	128 ± 2	136 ± 3	129 ± 3
DBP (mmHg)	78 ± 1	77 ± 1	81 ± 2	75 ± 2
MAP (mmHg)	95.3 ± 1	94 ± 2	99 ± 2	93 ± 2

Abbreviations: DBP, diastolic blood pressure; GLV, green leafy vegetable; MAP, mean arterial blood pressure; PLAC, placebo; PN, potassium nitrate; SBP, systolic blood pressure.

1 Data are mean ± SEM.

### BP and HR

**FIGURE 1:**
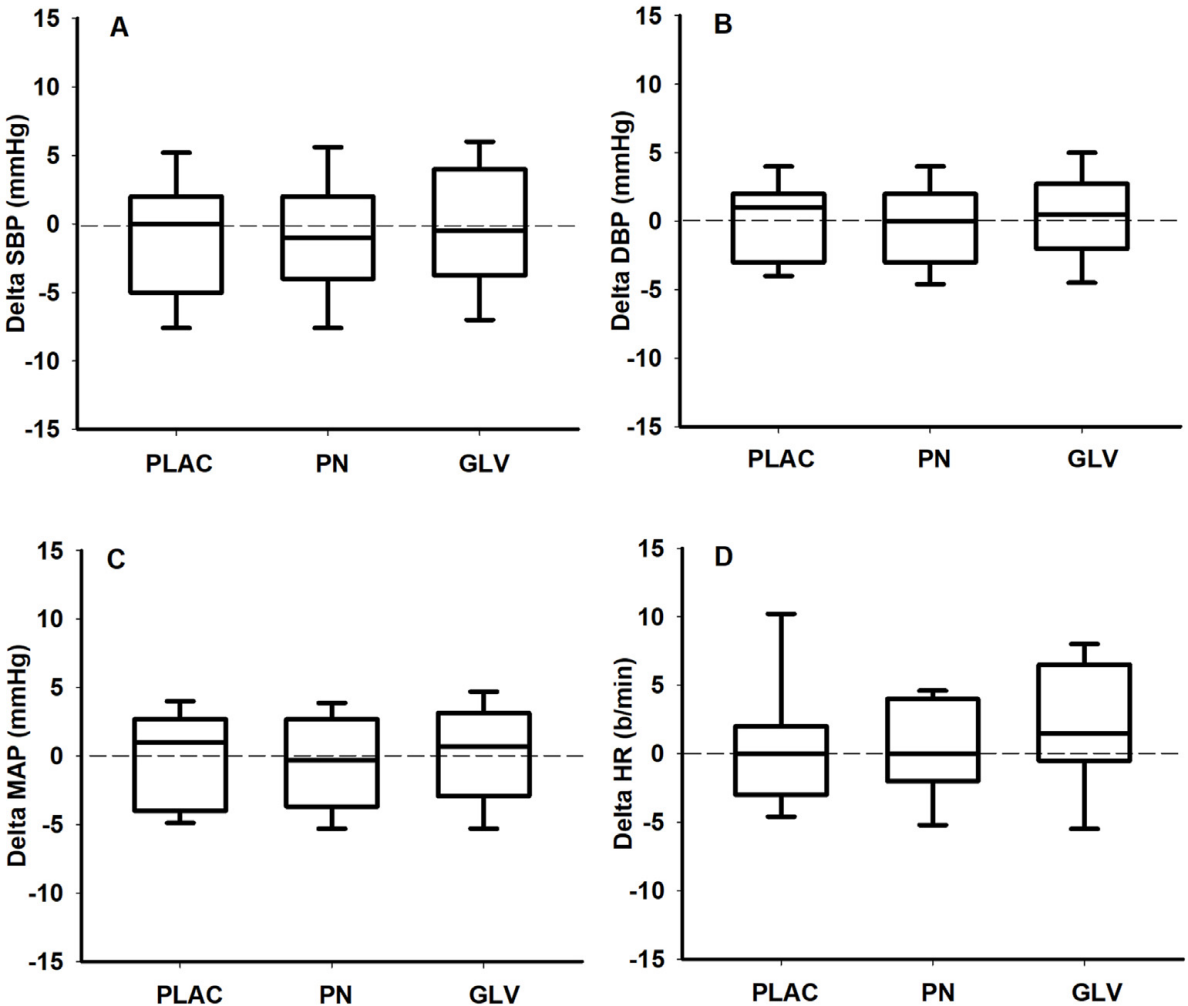
Changes (delta) in systolic blood pressure (SBP), diastolic blood pressure (DBP), mean arterial blood pressure (MAP), and heart rate (HR) before (pre) and after (post) the dietary intervention. GLV, green leafy vegetables group; PLAC, placebo group; PN, potassium nitrate group. Values are mean ± SD.

No changes were observed in SBP (PLAC: 129 ± 3; PN: 136 ± 3; GLV: 128 ± 3 mmHg), DBP (PLAC: 77 ± 1; PN: 81 ± 2; GLV: 75 ± 2 mmHg), mean arterial pressure (PLAC: 94 ± 2; PN: 99 ± 2; GLV: 93 ± 2 mmHg), and HR (PLAC: 68 ± 2; PN: 66 ± 1; GLV: 66 ± 2 b/min) after any of the dietary interventions compared with baseline (Figure 1).

### Salivary nitrate and nitrite

At baseline, salivary nitrate was significantly higher in the PN group, compared with the PLAC (*P* = 0.009) and GLV (*P* = 0.01) groups (Table 2). After the dietary intervention, a significant elevation of salivary nitrate was observed in the PN (*P* < 0.001) and GLV groups (*P* < 0.001) compared with baseline (Table 2). Salivary nitrate levels did not change in the PLAC group (*P* = 0.178) and were significantly lower compared with the PN (*P* < 0.001) and GLV (*P* < 0.001) groups after the dietary intervention. Salivary nitrate levels were similar among the PN and GLV (*P* = 0.848) after the dietary intervention (Table 2).

**Table 2:** Salivary nitrate, nitrite, pH, buffering capacity, ammonia, glucose and lactate levels, as well as plasma nitrate and nitrite (bottom) before (pre) and post (after) the dietary intervention.^1^

	Placebo (*n* = 23)		Potassium nitrate (*n* = 23)		Green leafy vegetables (*n* = 24)	
	Pre	Post	Pre	Post	Pre	Post
Saliva						
Nitrate (μmol/L)	175 ± 26	219 ± 36	470 ± 97	1788 ± 243^a^^,^^b^	333 ± 50	1905 ± 335^a^^,^^b^
Nitrite (μmol/L)	140 ± 21	172 ± 27	250 ± 50	500 ± 66^a^^,^^b^	152 ± 21	411 ± 65^a^^,^^b^
pH	7.33 ± 0.08	7.33 ± 0.11	6.94 ± 0.10^b^	7.40 ± 0.08^a^^,^^b^	7.19 ± 0.09	7.41 ± 0.08^a^^,^^b^
Buffering capacity (pH)	5.06 ± 0.23	5.36 ± 0.26	4.69 ± 0.23^b^	5.18 ± 0.27	4.57 ± 0.21^b^	5.25 ± 0.21^a^
Ammonia (mmol/L)	122 ± 16	140 ± 23	200 ± 18	157 ± 19^a^	137 ± 17	146 ± 17
Glucose (mmol/L)	0.11 ± 0.02	0.13 ± 0.03	0.18 ± 0.03	0.14 ± 0.04	0.15 ± 0.03	0.15 ± 0.03
Lactate (mmol/L)	0.18 ± 0.04	0.20 ± 0.05	0.24 ± 0.05	0.19 ± 0.04	0.21 ± 0.05	0.13 ± 0.02^a^
Plasma						
Nitrate (μmol/L)	28 ± 2.6	32 ± 2	36 ± 3	122 ± 11^a^^,^^b^	35 ± 2	128 ± 11^a^^,^^b^
Nitrite (nmol/L)	271 ± 40	340 ± 60	336 ± 50	459 ± 60^a^^,^^b^	315 ± 40	564 ± 140

1 Data are mean ± SEM.

a Statistical differences between pre and post in the same group.

b Statistical differences with the placebo group.

At baseline, salivary nitrite levels were higher in the PN group, but not statistically different compared with the PLAC (*P* = 0.054) and GLV (*P* = 0.694) groups (Table 2). After the dietary intervention, a significant elevation of salivary nitrite was observed in the PN (*P* = 0.003) and GLV groups (*P* = 0.002). Salivary nitrite levels did not change in the PLAC group (*P* = 0.166) and were significantly lower compared with the PN (*P* = 0.006) and GLV (*P* = 0.012) groups after the dietary intervention. Salivary nitrite levels were similar among the PN and GLV (*P* = 0.640) after the dietary intervention.

### Plasma nitrate and nitrite

At baseline, plasma nitrate was significantly higher in the PN group (*P* = 0.030) compared with the PLAC group, but not with the GLV group (*P* = 0.766) (Table 2). After the dietary intervention, a significant elevation of plasma nitrate was observed in the PN (*P* < 0.001) and GLV (*P* < 0.001) groups compared with baseline. Plasma nitrate levels did not change in the PLAC group (*P* = 0.107) and were significantly lower compared with the PN (*P* < 0.001) and GLV (*P* < 0.001) groups after the dietary intervention. Plasma nitrate levels did not differ among the PN and GLV groups after the dietary treatment (*P* = 0.655).

At baseline, plasma nitrite levels were not different between groups (*P* > 0.05) (Table 2). After the dietary intervention, a significant elevation of plasma nitrite was observed in the PN group (*P* = 0.016) compared with baseline. A similar increase was found in the GLV group, but it did not reach statistical significance (*P* = 0.082) compared with baseline. Plasma nitrite levels did not change in the PLAC group (*P* = 0.166) and were significantly lower compared with the PN group (*P* = 0.016), but not the GLV group (*P* = 0.187), after the dietary intervention. Plasma nitrite concentrations did not differ among the PN and GLV groups after the dietary treatment (*P* = 0.595).

### Salivary pH and buffering capacity

At baseline, salivary pH was significantly lower in the PN group (*P* = 0.008) compared with the PLAC group, but not to the GLV group (*P* = 0.127) (Table 2). After the dietary intervention, a significant elevation of salivary pH was observed in the PN (*P* < 0.001) and GLV (*P* = 0.030) groups. Salivary pH did not change in the PLAC group (*P* = 0.934) after the dietary intervention. Salivary pH was similar among the 3 groups (*P* > 0.05) after the dietary intervention.

At baseline, the buffering capacity of the PLAC group was significantly higher compared with the PN (*P* < 0.001) and GLV (*P* < 0.001) groups (Table 2). After the dietary intervention, a significant elevation of the buffering capacity was observed in the GLV group (*P* = 0.003) compared with baseline. No changes were found in the PLAC (*P* = 0.934) and PN groups (*P* = 0.147) after the dietary intervention. The buffering capacity was similar (*P* > 0.05) among the 3 groups after the dietary intervention.

### Salivary ammonia, glucose, and lactate

At baseline, salivary ammonia levels were significantly higher in the PN group compared with the PLAC group (*P* = 0.009), but not compared with the GLV group (*P* = 0.059) (Table 2). After the dietary intervention, a reduction in salivary ammonia was observed in the PN group compared with baseline, but it did not reach statistical significance (*P* = 0.068). No changes were observed in salivary ammonia levels in the PLAC (*P* = 0.199) and GLV (*P* = 0.355) groups after the dietary intervention. Salivary ammonia levels were similar among the 3 groups (*P* > 0.05) after the dietary intervention.

At baseline, salivary glucose levels were similar among the 3 groups (*P* > 0.05) (Table 2). After the dietary intervention, no significant changes (*P* > 0.05) were observed in salivary glucose in any of the 3 groups.

At baseline, salivary lactate levels were similar among the 3 groups (Table 2). After the dietary intervention, a significant reduction in salivary lactate was observed in the GLV group (*P* = 0.026). A reduction was also observed in the PN group, but it was not statistically different (*P* = 0.162). A small increase of salivary lactate, but not statistically significant (*P* = 0.378), was found in the PLAC group after the dietary intervention. A trend toward lower salivary lactate levels was found in the GLV group compared with the PLAC group after the dietary intervention, but it was not statistically significant (*P* = 0.074). Salivary lactate levels were similar between the GLV and PN groups (*P* = 0.177) after the dietary intervention.

### Associations between salivary nitrate and nitrite and other salivary biomarkers

At baseline, we found a negative moderate and significant association between salivary nitrate levels and pH (*r* = −0.49; *P* < 0.001) and buffering capacity (*r* = −0.43; *P* < 0.001). Salivary nitrite levels were also negatively associated with pH (*r* = −0.39; *P* = 0.001), and positively associated with glucose (*r* = 0.39; *P* = 0.001), lactate (*r* = 0.33; *P* = 0.005), and ammonia (*r* = 0.27; *P* = 0.027). We did not find associations between SBP, DBP, and mean arterial pressure with any salivary biomarker.

### Microbiome

#### Sequencing results

The high-throughput sequencing data are available within the NBC Sequence Read Archive as BioProject PRJNA1086240. Microbiome sequencing yielded an average 110,883 paired reads (66.8 Mbp) per sample, of which 93% passed the quality filters. After ASV clustering and chimaera removal, 91% of the filtered reads remained and were clustered in 6671 ASVs. In total, 65% of the remaining reads were taxonomically classified down to the species level, and 98% down to genus level.

#### α-Diversity and β-diversity

For α-diversity analyses, the observed diversity, Shannon index, and Simpson index measures were considered. The chosen measures did not significantly differ at baseline and no significant differences (*P* > 0.05) were observed in the paired Wilcoxon test when comparing the individuals before and after intervention at any taxonomic level.

**FIGURE 2:**
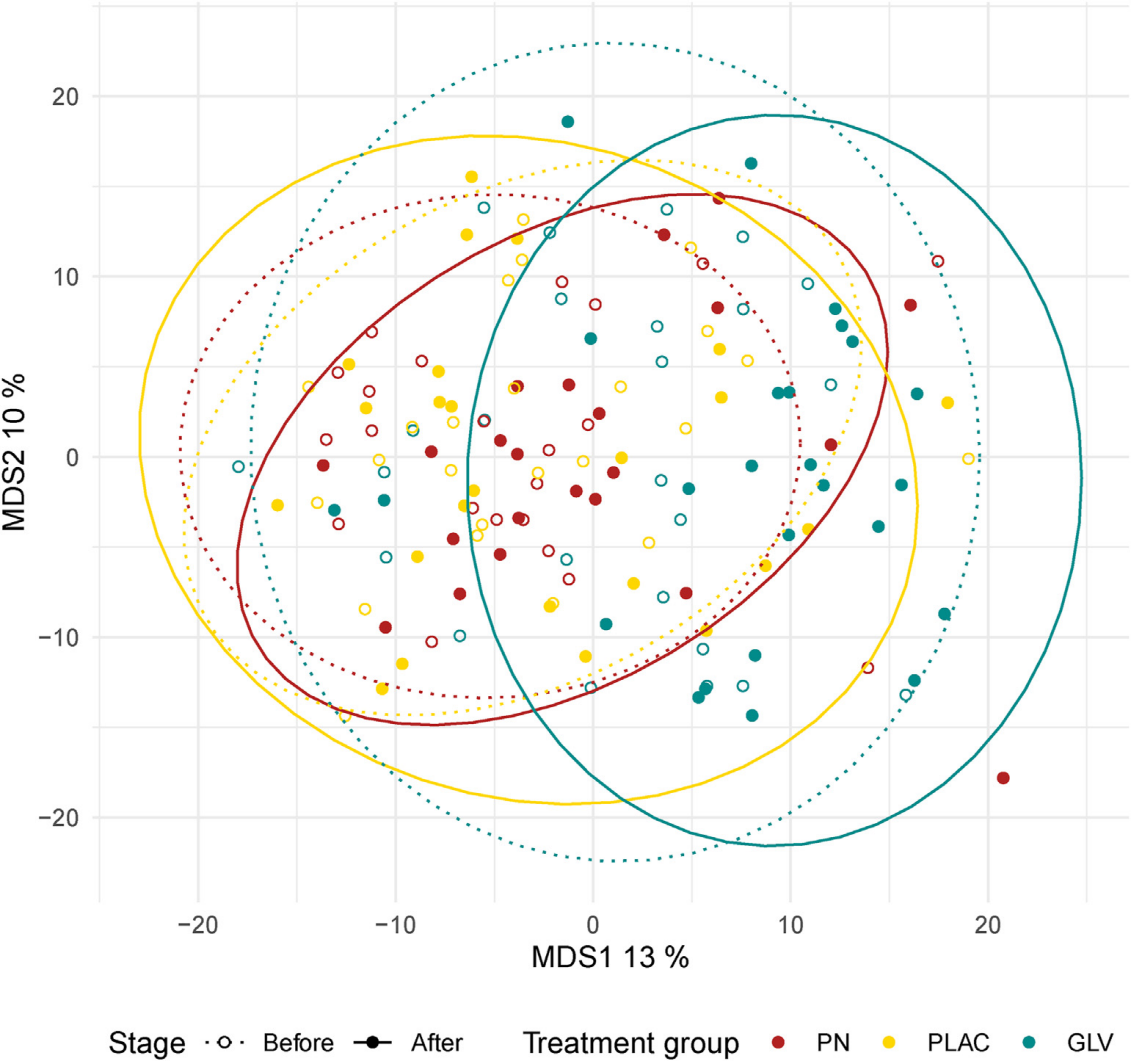
β-Diversity in the potassium nitrate (PN), placebo (PLAC), and green leafy vegetables (GLV) groups before (dotted lines) and after (continuous line) the dietary intervention.

β-Diversity analyses were based on Aitchison’s distance to account for compositionality. No difference was observed between the treatment groups prior to the treatment (*P* > 0.20 for all taxonomic ranks) (Figure 2). Significant changes (*P* < 0.05) were observed between pre- and post-intervention in the GLV group at species and family levels (Figure 2).

#### Differential bacterial abundance

**FIGURE 3:**
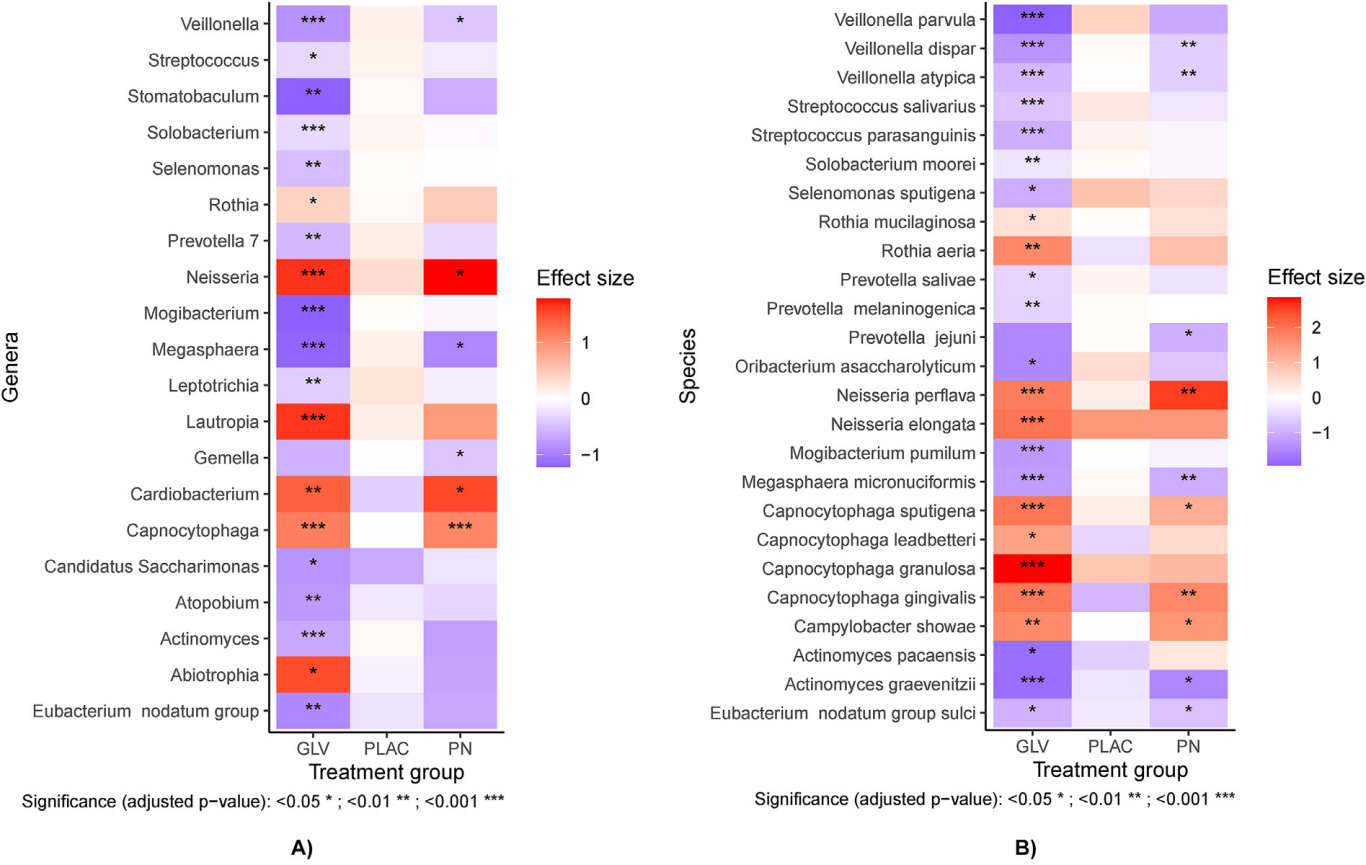
Heatmap of differentially abundant genera (A) and species (B) after the dietary intervention in the 3 groups (PLAC, placebo group; PN, potassium nitrate group; GLV, green leafy vegetables group). Only taxa that significantly changed in any of the groups are reported. The effect size corresponds to the effect of the treatment estimated with linear models.

At baseline, 13 taxa were identified as differentially abundant between any of 2 groups (Table 3). After the dietary intervention, the abundance of 6 genera (10 species) and 19 genera (24 species) were different in the PN and GLV groups, respectively, whereas no changes in bacterial abundance were observed in the PLAC group (Figure 3A, B). Regarding the higher taxonomic ranks, both the PN and GLV groups had a significant increase in phylum Pseudomonadota (*P* < 0.005) and a decrease in Bacillota (*P* < 0.001). Actinobacteria was also observed to decrease in the PN group (*P* = 0.03). Some of these bacterial changes including a reduction in the abundance of *Veillonella*, *Megasphaera*, *Actinomyces*, and *Eubacterium* species and an increase in *Neisseria*, *Capnocytophaga*, and *Campylobacter* were common in PN and GLV groups (nitrate-dependent) (Figure 3A). However, the GLV group showed some changes including an increase in *Rothia,* and a decrease in *Streptococcus, Prevotella, Actinomyces*, and *Mogibacterium* species that were not observed in the PN group (nitrate-independent).

**Table 3:** Differentially abundant taxa at baseline (prior to the dietary intervention) between any 2 groups.

Rank	Groups	Taxon	*P*	Size^1^
Order	GLV-PN	Bacteroidota	0.005	−0.60
Order	PLAC-PN	Corynebacteriales	0.004	2.00
Order	GLV-PN	Veillonellales.Selenomonadales	0.010	−0.64
Family	PLAC-PN	Corynebacteriaceae	0.004	2.08
Genus	PLAC-PN	Corynebacterium	0.003	2.04
Genus	GLV-PN	Corynebacterium	0.032	1.45
Genus	GLV-PN	Megasphaera	0.015	−1.22
Species	GLV-PN	Actinomyces_graevenitzii	0.019	−1.25
Species	PLAC-PN	Corynebacterium_matruchotii	0.004	2.08
Species	PLAC-PN	Parvimonas_micra	0.009	−2.28
Species	PLAC-PN	Veillonella_parvula	0.023	−1.97
Species	GLV-PN	Megasphaera_micronuciformis	0.013	−1.20

Abbreviations: GLV, green leafy vegetables group; PLAC, placebo group; PN, potassium nitrate group.

1 Size indicates the magnitude of the difference regarding the PN group.

#### Differential abundance of nitrate-reducing species

**FIGURE 4:**
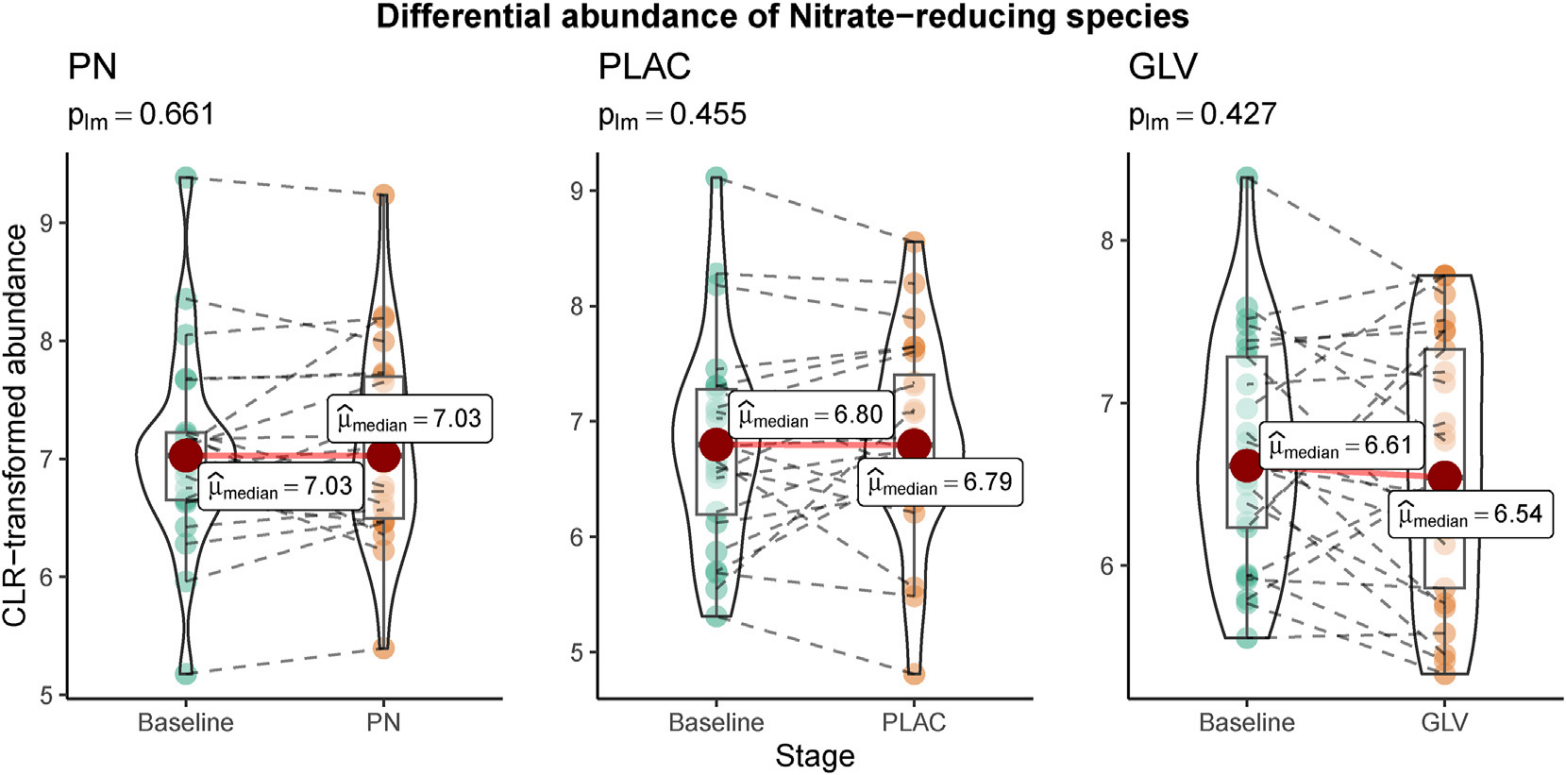
Differential abundance of nitrate-reducing species after the dietary intervention in the 3 groups (PLAC, placebo group; PN, potassium nitrate group; GLV, green leafy vegetables group). The *P* value corresponds to the raw *P* value obtained with linear models.

In our dataset, 13 of 28 described nitrate-reducing species were detected (Supplemental Table 1). At baseline, participants had similar abundance of oral nitrate-reducing species (*P* = 0.27). After the dietary intervention, the abundance of these 13 nitrate-reducing species was not significantly altered in any of the treatments (Figure 4).

#### Associations between bacterial abundance BP levels and salivary biomarkers

**FIGURE 5:**
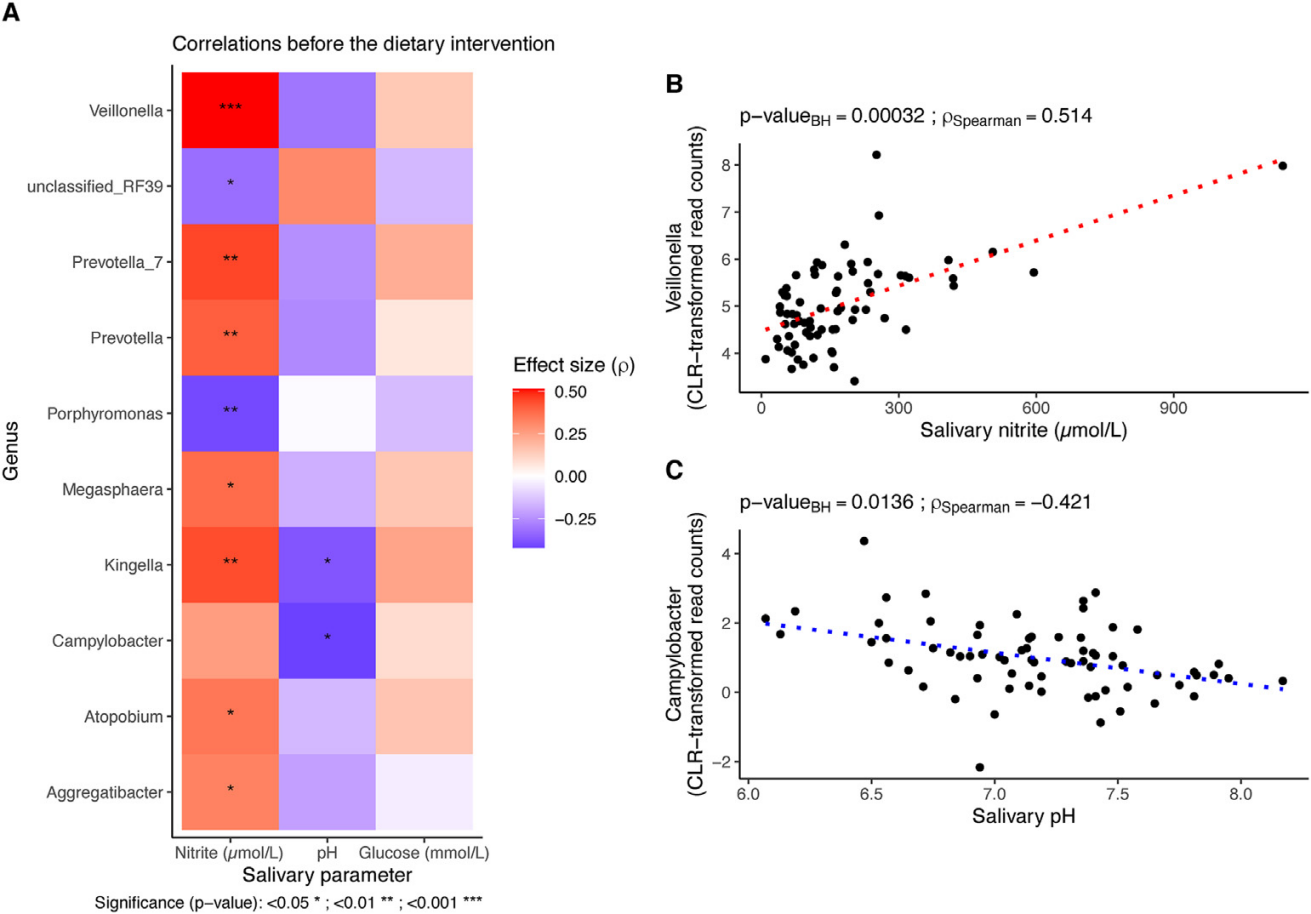
Correlations at baseline between bacterial genera and salivary parameters. Heatmap of effect size and significance of the correlation as estimated with Spearman’s correlation analysis (A). Correlations of *Veillonella* (B) and *Campylobacter* (C) abundance and salivary parameters.

Conducting exploratory analysis, we did not find significant associations between bacterial abundance before and after the dietary intervention and BP levels. On the other hand, taking the microbial data from all the participants together at baseline (*n* = 70), we found a positive and significant association between the microbial community and salivary nitrite (*P* = 0.001), pH (*P* = 0.023), and glucose (*P* = 0.044), which accounted for 11.9% of the variance. To further explore these associations, we computed correlations before the interventions between these variables and the bacterial genera. We identified several significant correlations (Figure 5A), especially with salivary nitrite. Some genera, including *Prevotella* and *Veillonella* (Figure 5B) had a positive correlation with salivary nitrite, whereas genus *Porphyromonas* had a negative correlation. Campylobacter and Kingella showed a significant negative correlation with salivary pH (Figure 5C).

## Discussion

To the best of our knowledge, this is the first study to compare the impact of dietary nitrate in different forms (GLV and PN) on the oral microbiome. Our findings partially support the primary hypothesis, indicating that dietary nitrate in both forms alters the composition of the oral microbiome, leading to a higher abundance of *Neisseria* species and lower abundance of *Veillonella* species, likely indicating nitrate-dependent changes. However, contrary to our hypothesis, the GLV group showed an increase in *Rothia* species and a reduction in *Streptococcus*, *Prevotella*, *Actinomyces*, and *Mogibacterium* species, changes not observed in the PN group. These changes are likely nitrate-independent.

Previous studies administering nitrate in the form of beetroot juice for 1 and 2 wk in healthy individuals [3] and patients with hypercholesterolaemia [4], respectively, reported similar microbial changes (an increase in *Rothia* and *Neisseria* and a reduction in *Prevotella* and *Veillonella* species) comparable with those found in the GLV group. Using a similar approach (beetroot juice supplementation × 7 d), Burleigh et al. [5] found an increase in *Neisseria* and a reduction in *Prevotella* and *Actinomyces* species on the tongue dorsum of healthy individuals. The ingestion of lettuce juice, rich in nitrate, for 14 d increased the abundance of *Rothia* and *Neisseria* species in individuals with periodontal disease [6]. According to our results, the observed increase in *Rothia* species and decrease in *Prevotella*, and *Actinomyces* species in studies providing nitrate in the form of beetroot and lettuce juice may be nitrate-independent, whereas increases in *Neisseria* species and reductions in *Veillonella* may be nitrate-dependent changes.

In this study, we identified 13 of 28 species reported as nitrate-reducers in previous studies [7,8,32]. This list is based on in vitro studies, where *Veillonella* and *Rothia* species were identified as the most abundant nitrate-reducing genera [7,8,32]. We found that the abundance of identified nitrate-reducing species was not altered in any of the dietary interventions. This contrasts with our initial hypothesis, which suggested that higher dietary nitrate intake could increase the abundance of nitrate-reducing bacteria. However, it is worth to note that changes in bacterial abundance may not necessarily reflect alterations in functionality [33]. Consequently, future studies should integrate metagenomic and functional analyses (that is, metatranscriptomics) to quantify both the abundance and activity of nitrate-reducing bacteria. This approach would provide a more comprehensive understanding of the impact of dietary compounds on the oral microbiome. Additionally, given the recent advancements in next-generation techniques, it may become feasible to identify other bacterial species with nitrate-reducing activity in the near future, potentially altering the list of bacteria used in our analysis.

Analyzing the baseline data from all the participants, we observed a positive and significant association between some nitrate-reducing species, such as *Veillonella* and *Prevotella,* and salivary nitrite levels. Although this association may suggest a potential contribution of these bacteria to salivary nitrite levels, some caution is needed when interpreting these associations. The activity of immune cells can also impact nitrate and nitrite levels in saliva. The activation of immune cells in periodontal disease can upregulate nitric oxide synthesis, which is rapidly converted into nitrate and nitrite [34]. Consequently, an elevation of nitrite in saliva could be expected in individuals with periodontal disease. Our analysis also revealed a negative and significant association between salivary pH, nitrate, and nitrite levels at baseline, suggesting potential indicators of poor oral health in individuals with low salivary pH and high nitrate and nitrite levels. However, it is important to consider that other sources of nitrate, such as toothpaste, could influence the concentration of nitrate and nitrite in this study. We should also be cautious when interpreting these associations given the limited sample size of this study. Future research should investigate oral nitrite metabolism in different oral health conditions. This knowledge can be useful to identify novel strategies for managing and treating periodontal disease including diet [11].

Salivary pH is an important factor for preserving oral health [10]. A pH below 7 can increase the risk of oral disease [10]. Interestingly, the consumption of dietary nitrate in both forms, PN and GLV, increased the salivary pH and this was accompanied by a decrease in *Veillonella* and *Prevotella* species. Previous studies have also reported a similar increase in salivary pH after dietary nitrate consumption [3,5,35]. Additionally, dietary nitrate in the form of GLV increased the salivary buffering capacity and reduced salivary lactate, both of which are positive changes associated with resilience against oral disease [36]. This effect is likely nitrate-independent, because it was not observed in the PN group. The presence of other bioactive compounds in GLV may offer additional benefits for managing periodontal disease compared with PN. The higher abundance of *Rothia* species, particularly *R. mucilaginosa*, induced by GLV may contribute to these changes, given their ability to metabolize acids such as lactate [37]. Furthermore, better oral health outcomes have been reported in patients with periodontal disease after increased dietary nitrate intake in the form of lettuce juice [6], highlighting the potential role of dietary interventions, including high-nitrate vegetables, in oral health management.

It is important to note that the analysis of the oral microbiome and salivary biomarkers at baseline was conducted after a 2-wk period of restricting the consumption of high-nitrate vegetables. However, considering that the standard consumption of dietary nitrate is typically <100 mg/d [38,39], we believe that this change had minimal impact on the composition of the oral microbiome. In fact, another interesting finding of this study was that a diet rich in low-nitrate vegetables (PLAC) had a small effect on the oral microbiome and salivary biomarkers, despite containing other potential bioactive compounds. This suggests that nitrate is an important compound in vegetables for eliciting a prebiotic effect. Our study indicates that a daily intake of ≥300 mg/d, especially in the form of GLV, may be necessary to trigger the prebiotic effects of nitrate.

Besides oral health, a substantial number of studies, but not all [18,19,[40], [41], [42], [43]], in healthy and hypertensive individuals have shown a blood pressure–lowering effect after acute and prolonged consumption of dietary nitrate [14,17,[44], [45], [46], [47], [48], [49], [50]]. The DINO study was originally conducted to investigate the effectiveness of dietary nitrate in the form of PN and GLV on BP levels. In the cohort of participants from the DINO study that we investigated, we observed no changes in BP in either the PN or GLV groups. This finding is consistent with the results reported in the original DINO study [20]. Currently, it is difficult to explain the lack of BP reduction compared with other studies and further research is needed to clarify this discrepancy [14,17,[44], [45], [46], [47], [48], [49], [50]].

We did not observe an association between oral bacterial abundance, particularly nitrate-reducing species, and BP levels. Although this finding did not align to our initial hypothesis, it is consistent with a previous study by Goh et al. [27], which reported an association between nitrate-reducing species and SBP level in normotensive individuals, but not in hypertensive ones. Future studies investigating the link between the oral microbiome and BP must include oral health examinations because periodontal disease can profoundly influence the composition of the oral microbiome and bioavailability of nitrate and nitrite in the oral cavity.

This study had some limitations that are worth to comment. First, we analyzed a small cohort of the DINO study because of constrains of saliva availability. Second, the estimated energy intake was slightly higher (40 kcal/d = 1500 kcal for the 5-wk intervention period) in the low-nitrate vegetable group (PN and PLAC). However, this did not have an impact in body weight, and our dietary approach was robust to assess the effect of dietary nitrate on the oral microbiome. Third, we observed a low salivary pH in the PN group at baseline; however, it is difficult to indicate whether these differences could be attributed to the oral health status.

In conclusion, dietary nitrate intake in both natural (GLV) and pharmacological (PN) forms can alter the oral microbiome. However, GLV have a more pronounced effect on altering the composition of the oral microbiome compared with PN. This could be related to the presence of other bioactive compounds in GLV, which could induce nitrate-independent changes. Interestingly, oral microbial changes induced by GLV demonstrated a positive effect on regulating salivary pH, buffering capacity, and salivary lactate levels, underscoring their potential for managing and treating periodontal disease. On the other hand, oral microbial changes were not associated with a reduction in BP levels.

## Acknowledgments

We thank Eva Wallgren, Victoria Boström Nilsson, and Merja Heinonen at the Department of Medicine and Karolinska University Hospital for excellent support with executing the study and Carina Nilén and Annika Olsson at the Department of Physiology and Pharmacology (Karolinska Instute, Sweden) for expert technical assistance.

## Author contributions

The authors’ responsibilities were as follows – MLS, EW, JOL: designed the research; MLS: conducted the research; MLS, LdT, ZB, PCA, AR, GM, TG, RB: analyzed the data; LdT, RB, ZB, MH, RB: wrote the article; TG, AR, AB: wrote microbiome analyses sections of the article; MLS, EW, JOL: had primary responsibility for final content; and all authors: read and approved the final manuscript.

## Conflict of interest

JOL and EW are co-inventors on a patent application related to the therapeutic use of inorganic nitrate. All other authors report no conflicts of interest.

## Funding

The DINO study was funded by the Af Jochnick Foundation and the AxFood Foundation for their contribution of vegetables and deliveries throughout the study period. Metagenomic, bioinformatics, and biochemistry analyses of this study were funded by the University of Plymouth (UK) and the Institut de Recerca Biomedica (IRB, Barcelona, Spain).
