# Supplementary material for: The Effect of Dietary Nitrate on the Oral Microbiome and Salivary Biomarkers in Individuals with High Blood Pressure
Source: J Nutr. 2024 Jul 16;154(9):2696–706. doi: 10.1016/j.tjnut.2024.07.002 (PMC11393165; doi:10.1016/j.tjnut.2024.07.002)
Supplement: Multimedia component 1 [file mmc1.docx]

**Supplementary data**

**The effect of dietary nitrate on the oral microbiome and salivary biomarkers in individuals with high blood pressure.**

**Lisa du Toit et al.**

**Supplementary data 1: DNA extraction and sequencing**

The ZymoBIOMICS® Gut Microbiome Standard (ref #D6331) was used as a positive control and treated as the other samples. DNA extraction was performed with the ZymoBIOMICS 96 MagBead DNA kit (ref #D4302, ZymoResearch), using the FastPrep-96 and 32-PurePrep (MolGen) as described below.

A deep-well plate was prepared containing the following solutions of the kit: columns 1 and 7: 600 μl MagBinding buffer, 25 μl of MagBinding Beads; columns 2 and 8: 500 μl MagBinding buffer; columns 3 and 9: 500 μl MagWash 1; columns 4, 5, 10 and 11: 900 μl MagWash 2, and column 6 and 12: 50 μl ZymoBIOMICS™ DNase/RNase Free Water.

Saliva pellets were resuspended in 800μl Lysis solution of the kit and 800μl were transferred into a ZymoResearch BashingBead™ Lysis Tube. These tubes were agitated in FastPrep-96 at max speed (1,800 rpm) for 5 minutes and then centrifuged at 5,000 g for 5 minutes. 200 μl of the supernatant were added to columns 1 and 7 of the above mentioned prepared deep-well plate.

The plate was introduced in the 32-PurePrep machine, with the following configuration:

1. STEP 1/WELL 1: LyseBind. Mix time: 10 minutes. Magnet: 60 seconds. Wait Time: 0 minutes. Volume: 825 μl. Mix Speed (1-10): 10. Temperature: OFF.
2. STEP 2/WELL 2: MagBind. Mix time: 1 minute. Magnet: 30 seconds. Wait Time: 0 minutes. Volume: 500 μl. Mix Speed (1-10): 8. Temperature: OFF.
3. STEP 3/WELL 3: MagWash1. Mix time: 1 minute. Magnet: 30 seconds. Wait Time: 0 minutes. Volume: 500 μl. Mix Speed (1-10): 8. Temperature: OFF.
4. STEP 4/WELL 4: MagWash2. Mix time: 1 minute. Magnet: 30 seconds. Wait Time: 20 minutes. Volume: 900 μl. Mix Speed (1-10): 8. Temperature: OFF.
5. STEP 5/WELL 5: MagWash2. Mix time: 1 minute. Magnet: 30 seconds. Wait Time: 0 minutes. Volume: 900 μl. Mix Speed (1-10): 8. Temperature: OFF.
6. STEP 6/WELL 6: Elution. Mix time: 10 minutes. Magnet: 60 seconds. Wait Time: 0 minutes. Volume: 50 μl. Mix Speed (1-10): 5. Temperature: OFF.
7. STEP 7/WELL 5: Discard. Mix time: 1 minute. Magnet: 0 seconds. Wait Time: 0 minutes. Volume: 900 μl. Mix Speed (1-10): 8. Temperature: OFF.

After this step, the eluted samples at columns 6 and 12 were transfer to a 96 well plate to proceed to 16S amplicon sequencing.

The V3-V4 region of the bacterial 16S ribosomal RNA gene was amplified using the following universal primers in a limited cycle PCR: V3-V4-Forward (5′-TCGTCGGCAGCGTCAGATGTGTATAAGAGACAGCCTACGGGNGGCWGCAG-3′) and V3-V4-Reverse (5′-GTCTCGTGGGCTCGGAGATGTGTATAAGAGACAGGACTACHVGGGTATCTAATCC-3′). Then full-length Nextera adapters with barcodes for multiplex sequencing were added in a second PCR step, resulting in sequencing ready libraries. Sequencing was performed in the Illumina MiSeq with 2 × 300 bp reads using v3 chemistry at the Genomics facility of the Centre for Genomic Regulation (CRG, Barcelona). Two bacterial mock communities from the BEI Resources of the Human Microbiome Project (HM-276D and HM-277D) were amplified and sequenced in the same manner as all other samples. Negative controls of PCR amplification were also included in parallel, using the same conditions and reagents.

**Supplementary data 2: Microbial statistical analyses**

Microbial community analyses were performed with R v4.3.1 and Rstudio v2023.06.0.421. R package dada2 v1.12.1 (43) was used for sequence quality filtering, with parameters ‘truncLen = c(270, 225), trimLeft = 10 minLen = 50, maxEE = 8, maxN = 0’. Dada2 was also used for ASV clustering, chimera removal and taxonomic assignment of ASVs with database SILVA nr99 v138.1 (44). Phyloseq v1.44.0 (45) was then used to analyse the abundance tables. To reduce noise, ASVs present in less than 20 samples were removed, considering only samples with more than 50 reads of that given ASV. Because of the compositional nature of metagenomic data (46), abundance tables were 0-replaced using the CZM method with R package zCompositions v1.4.0.1 (47) and then CLR-transformed with package CoDaSeq v0.99.6. Principal Component Analysis (PCA) were performed with the *prcomp* function in package stats v4.3.1, and correlation of metadata with the principal components was tested with a Welch’s F-test. Alpha diversity measures were compared using Wilcoxon and Wilcoxon signed-rank tests. Beta diversity analyses were based on a PERMANOVA test on Aitchison’s distance, calculated with package robCompositions v.2.3.1 (48). Differential abundance analyses were performed using mixed-effect linear models with R packages lmer4 v1.1.33 (49). Sequencing batch, sex and age were passed on to the linear models as fixed effects in all cases. When comparing paired samples, the individual was added as a random effect. P-values for the multiple tests were adjusted using the Benjamini-Hochberg correction (FDR correction), and only correlations with an adjusted p-value < 0.05 were considered significant and reported. For the analysis of nitrate-reducing taxa, read counts of nitrate-reducing species and genera were identified following the classification from Goh et al*.* (38), and the summed read counts were considered as a single taxon for the downstream analyses. Links between bacterial taxa and salivary biomarkers were assessed with a Spearman’s correlation analysis.

**Supplementary table 1:** Abundance of nitrate-reducing genera (left) and species (right) at baseline (TRC: total read counts; RRC: Relative read counts).

| **Nitrate-reducing genera** | **TRC** | **RRC** | **Nitrate-reducing species** | **TRC** | **RRC** |
| --- | --- | --- | --- | --- | --- |
| *Actinomyces* | 337992 | 2.63% | \| *Capnocytophaga sputigena* \| \| --- \| \|  \| | 33611 | 0.26% |
| *Fusobacterium* | 565653 | 4.41% | *Corynebacterium matruchotii* | 12921 | 0.10% |
| *Granulicatella* | 108439 | 0.85% | *Haemophilus parainfluenzae* | 813999 | 6.34% |
| *Haemophilus* | 1002048 | 7.81% | *Neisseria mucosa* | 27888 | 0.22% |
| *Leptotrichia* | 574861 | 4.48% | *Neisseria perflava* | 757894 | 5.91% |
| *Neisseria* | 1067773 | 8.32% | *Prevotella melaninogenica* | 1280287 | 9.97% |
| *Porphyromonas* | 780500 | 6.08% | *Prevotella salivae* | 316527 | 2.47% |
| *Prevotella* | 1021580 | 7.96% | *Rothia dentocariosa* | 21471 | 0.17% |
| *Veillonella* | 2089440 | 16.28% | *Rothia mucilaginosa* | 146614 | 1.14% |
|  |  |  | \| *Selenomonas noxia* \| \| --- \| \|  \| | 3268 | 0.03% |
|  |  |  | \| *Veillonella dispar* \| \| --- \| | 698586 | 5.44% |
|  |  |  | *Veillonella parvula* | 173397 | 1.35% |
|  |  |  | *Veillonella atypica* | 804500 | 6.27% |
